# The Relationship between Physical Activity and Screen Time with the Risk of Hypertension in Children and Adolescents with Intellectual Disability

**DOI:** 10.1155/2017/1940602

**Published:** 2017-11-02

**Authors:** Justyna Wyszyńska, Justyna Podgórska-Bednarz, Katarzyna Dereń, Artur Mazur

**Affiliations:** ^1^Institute of Physiotherapy, Medical Faculty, University of Rzeszów, Rzeszów, Poland; ^2^Institute of Nursing and Health Sciences, Medical Faculty, University of Rzeszów, Rzeszów, Poland

## Abstract

**Introduction:**

Children and adolescents with intellectual disability (ID) have significantly lower levels of physical activity compared to their peers without ID. Association between the level of physical activity and screen time with hypertension (HPT) in children and adolescents with ID has not been reported yet.

**Aim:**

To assess the relationship between the level of physical activity and screen time with the prevalence of HPT in students with ID.

**Material and Methods:**

The study group consisted of 568 children with ID aged 7 to 18. The control group matched for age and gender consisted of 568 students without ID. Blood pressure (BP), body mass and height, level of physical activity, and screen time were assessed.

**Results:**

The level of physical activity in the study group was significantly lower than in the control group (score 1.99 versus 3.02, resp., in* Physical Activity Questionnaire*). The risk of HPT in the students with ID with low levels of physical activity was more than 4 times higher (OR = 4.40) and more than 2 times higher when screen time was ≥2 h/day.

**Conclusion:**

Low level of physical activity and long screen time were associated with significantly higher HPT risk among children and adolescents with ID.

## 1. Introduction

Low level of physical activity is responsible for more than five million deaths globally per year, being one of the most important factors for the risk of noncommunicable diseases [1]. Epidemiological evidence has revealed a consistent, temporal, and independent relationship between low level of physical activity and HPT [2]. As the prevalence of HPT is increasing worldwide, primary prevention of HPT has become an important public health initiative. Physical activity is commonly recommended as an important element of lifestyle modification, which may help to prevent HPT [3].

Data show that 80% of children and adolescents in 105 countries do not achieve the recommended level of physical activity, that is, at least 60 minutes of moderate to vigorous physical activity daily [4]. In addition, high number of children and adolescents spend more than 4 hours a day on sedentary activities, although less than 2 hours a day are recommended for screen-based entertainment (TV viewing, computer, and videogame usage) [5, 6]. Research results showed that TV viewing and screen time were associated with unfavorable body composition, decreased fitness, lowered scores for self-esteem, and prosocial behavior [7] as well as higher systolic BP and diastolic BP in children and adolescents [8, 9].

Even lower levels of physical activity are observed among the population of children and adolescents with ID. Numerous studies indicate that youth with ID compared to the peers without ID have significantly lower levels of physical activity [10–13]. This is not only due to the limitations associated with ID and its consequences, but also due to numerous environmental barriers, financial limitations, lack of social support, lack of motivation, low levels of awareness, and self-efficacy [14]. Low level of physical activity and high level of sedentary behaviors in combination with genetic predisposition, level of ID, improper diet, and medication increase the chances of obesity, which is one of the risk factors for HPT [15].

The incidence of HPT in children and adolescents with ID is significantly higher than their peers without ID [16, 17]. This is a serious public health problem, as the results of epidemiological studies indicate that HPT in childhood is a factor that determines the incidence of HPT in later life [18]. In addition, HPT in adolescence is strongly correlated with an increase in morbidity and mortality rates in adulthood [19]. In the case of children and adolescents with different types and levels of ID, lack of activity is particularly disadvantageous as physical activity is also a part of the process of education, development, and rehabilitation [20].

We have not found any reports on the relationship between the level of physical activity and screen time with the prevalence of HPT in the developmental age population with ID.

## 2. Purpose of the Paper

Therefore, the aim of the study was to assess the relationship between the level of physical activity and screen time with prevalence of HPT in children and adolescents with ID.

## 3. Material and Method

The study was approved by the Bioethics Committee at the Medical Faculty of the University of Rzeszów. A written consent from all participants was obtained prior to the study. Information regarding the birth date and the participants' level of ID was obtained from the medical files kept by the school nurses (a certificate of disability issued by the Disability Evaluation Board, at the Psychological and Pedagogical Counseling Center). The anthropometric measurements were carried out at schools, in nurses' offices. All measurements were taken between 10:00 a.m. and 12:00 a.m.

### 3.1. Participants

The study group consisted of students with ID aged 7–18, who were enrolled in public special education schools in southeastern Poland in the school year 2013/2014. The invitation to participate in the study was sent to all parents of children attending special schools in southeastern Poland (*n* = 2282). Approximately 908 parents gave consent to participate in the study. Of those, 340 were excluded from the study due to age: <7 yrs or >18 yrs (*n* = 59), taking medication affecting BP (*n* = 21), functional state that does not allow for self-maintenance of standing position (*n* = 16), lack of cooperation or strong anxiety of the examination (*n* = 28), being absent from school on test days (*n* = 53), and failure to return or incompletely completed survey (*n* = 163). Ultimately, the study group consisted of 568 students.

The control group consisted of students without ID attending randomly selected primary, middle and secondary schools in southeastern Poland. The invitation to participate in the study was sent to 3500 parents of students from the randomly selected schools. 1833 parents agreed for the child's participation in the study. Of these, 568 students were excluded due to age: <7 yrs or >18 yrs (*n* = 2), taking medication affecting BP (*n* = 7), functional state that does not allow for self-maintenance of standing position (*n* = 1); lack of desire to participate in study or strong anxiety before the examination (*n* = 6), being absent from school on test days (*n* = 77), and failure to return or incompletely completed survey (*n* = 475). Out of 1265 tested students, 568 students were selected to the control group (age and sex adjustment). The students sampling to the control group was performed using Statistica software, sampling without replacement.

### 3.2. Body Height and Body Mass

Body height was measured with an accuracy of 0.1 cm using the PORTSTAND 210 portable stadiometer. The measurement was performed under standard conditions, in an upright position, barefoot.

The body mass was assessed with the accuracy of 0.1 kg using a body composition analyzer (BC-420 SMA, Tanita). The subjects were weighed in underwear, barefoot.

Body mass index (BMI) was calculated by dividing the body mass (in kg) by the height (in meters) squared (BMI = body mass/body height^2^). Based on BMI values, the BMI centile of individual students was calculated. BMI centile charts specific for age, sex, and body height (which were developed in the framework of the Polish project entitled “Developing standards of blood pressure in children and adolescents in Poland, OLAF”) were used [21]. Based on the percentile values, the subjects were assigned to the following categories: obesity (BMI ≥ 95th percentile), overweight (BMI ≥ 85th percentile and <95th percentile), healthy weight (BMI < 85th percentile and ≥5th percentile), and underweight (BMI < 5th percentile) [22].

### 3.3. Arterial Blood Pressure

BP was measured three times with an aneroid sphygmomanometer in accordance with the current NHBPEP guidelines [23]. According to recent research result, the aneroid device has better accuracy than the digital device as compared to mercury sphygmomanometer and should be used for proper and better management. It has been found that the agreement between the mercury and aneroid sphygmomanometer in classifying hypertension is very high (kappa = 0.881, *p* < 0.001). This suggests the greater ability of the aneroid instruments in classifying an individual as hypertensive or normotensive. In the validity analysis, it has been studied whether the aneroid and digital devices produced accurate results (one that lacked systematic error) by calculating sensitivity, specificity, positive, and negative predictive value, the mercury device being considered as a gold standard. All the indicators showed better results for aneroid device in comparison to the digital device [24].

BP was measured by trained staff predominantly by using aneroid sphygmomanometers recalibrated as needed by bioengineering services. Based on SBP and DBP, blood pressure centiles were estimated. The centile charts developed in the framework of the OLAF project were used [25]. Based on the percentile values obtained, the subjects were assigned to the following categories: normotension (BP was under the 90th percentile), prehypertension (BP ≥ 90th percentile or ≥120/80 mmHg, but <95th percentile for age, sex, and height), and HPT (BP ≥ 95th percentile and/or the use of antihypertensive medication) [23].

### 3.4. Physical Activity Level and Screen Time

*Physical Activity Questionnaire for Older Children* (PAQ-C) and* Physical Activity Questionnaire for Adolescent* (PAQ-A) were used to assess the physical activity of children and adolescents. The questionnaires serve to assess the overall level of physical activity in children and adolescents during a school year. Both questionnaires include questions about physical activity undertaken in the past 7 days. The questionnaires cover questions about sports, games, and physical activity at school and during leisure, including weekends. Every question is scored on a 5-point scale (1 to 5 points), with higher scores representing higher level of physical activity [26]. The self-report PAQ-C and PAQ-A are valid, feasible, and cost-effective tools to evaluate physical activity in youth and are regarded as some of the most suitable self-report tools for examining physical activity in these populations [27]. These questionnaires have been used to test for multiple psychometric properties, that is, item and scale, internal consistency, test-retest reliability, convergent validity, construct validity, and sensitivity to gender and age differences [28–31]. All of these properties have been reported as acceptable to good. The PAQ-A has been validated against objectively measured physical activity (such as accelerometers) (*r* = 0.33–0.63) and other self-reported measures of physical activity (*r* = 0.73) [31, 32]. Construct validity of the PAQ-C has been demonstrated through correlations with aerobic fitness (*r* = 0.28) and perceptions of athletic competence (*r* = 0.48) [28]. Acceptable test-retest reliability and internal consistency have also been demonstrated for the PAQ-C and PAQ-A [30, 32].

Based on the value of the final PAQ score, the subjects were classified into three groups. Scores ≤ 2.3, from 2.4 to 3.7 and ≥3.8 pts represented the categories “low,” “moderate,” and “high” level of physical activity, respectively [30].

Total screen time (hours per day) was calculated as the average time spent on the following activities: TV/videos viewing, computer usage (both entertainment and homework), and using video games in week days and weekends.

Total time spent on sedentary activities was estimated. According to the international screen time recommendations, screen time was divided into two categories: (i) <2 h/day and (ii) ≥2 h/day [5].

Information about physical activity and screen time in younger participants without ID (7 to 13 yrs) was reported by their parents as evidence indicates that children under 9 years of age have no cognitive ability to accurately recall or evaluate their physical activity [33]. All necessary data relating to participants with ID were collected from their parents or guardians.

### 3.5. Data Analysis

Descriptive statistics was used in the analysis: mean/standard deviation (SD). Mann-Whitney* U* test (for binary variables) and Kruskal-Wallis test (for variables above two categories) were used to examine differences between independent quantitative variables. The selection of nonparametric tests resulted from lack of normality of variables (verified by Kolmogorov-Smirnov and Shapiro-Wilk tests). To examine the significance of differences between nominal variables, the independence test *χ*2 was used. The odds ratio (OR) was also used to discuss the frequency of a given variable. In order to determine the factors influencing the occurrence of hypertension in the study group multinomial logistic regression was used (progressive step method). The significance level was adopted at *p* < 0.05. Calculations were performed using IBM SPSS Statistics 20.

## 4. Results

The analyses were carried out for 568 students with ID (283 girls, 285 boys), aged 7–18, as well as 568 controls without ID, matched for age and sex. The distribution of age, sex, anthropometric data, level of physical activity, and screen time in both groups of subjects is presented in Table 1. The level of physical activity in the study group was significantly lower than in the control group (1.99 vs. 3.02 PAQ score, resp.). Most students with ID (70.4%) had low levels of physical activity, and none of the students attained high physical activity level. Screen time during school days in children and adolescents with ID was significantly longer than in their peers without ID (3.38 versus 2.27 h/day), while on weekends it was comparable in both groups.

Table 2 shows the values of the odds ratios for the prevalence of HPT, depending on the level of physical activity. The students with ID with low physical activity level have a greater risk of HPT than the students with moderate levels of physical activity (OR = 4.40). This relationship was stronger in the control group (OR = 5.04); however, the reference group in this case were the subjects with high level of physical activity (not moderate as in the case of study group).

Table 3 presents the values of odds ratios of the prevalence of HPT in relation to screen time. The students with ID whose screen time was ≥2 h/day during both school days and weekends were more likely to have HPT (OR = 2.21 and 2.24, resp.) than students whose screen time was <2 h/day. There was a slightly higher risk of HPT (OR = 2.74) in the group of students without ID who had a screen time ≥2 h/day during school days.

The findings presented in Table 4 are related only to the children and adolescents with ID. The results of multinomial logistic regression indicated that boys, the adolescents aged 14–18 yrs, the individuals with obesity, and those with screen time ≥2 h/day had higher risk of HPT; however, obesity proved to be the strongest factor (Table 4).

## 5. Discussion

Many studies have examined the association between physical activity, screen time, and HPT in children and adolescents [34–36], but to date, there have been no data on children and adolescents with ID. Therefore, the purpose of this study was to analyze the relationship between the level of physical activity and screen time with the prevalence of HPT in children and adolescents with ID and comparison with their peers without ID. In this study, we demonstrated that students with ID with low physical activity level were over 4 times more likely to develop HPT compared to the subjects with moderate levels of physical activity. Screen time also proved to be important predictor of HPT. We showed that the risk of HPT in subjects with ID who spent at least 2 hours per day on sedentary activities was more than twice as high as in the subjects with shorter time. According to our knowledge, the results presented in our research are the first concerning the relationship between the physical activity and screen time with prevalence HPT in children and adolescents with ID. Therefore, it is impossible to compare our results with the results of other authors. However, we conducted multivariate analysis to find out which factors have the strongest impact on the prevalence of HPT in children and adolescents with ID. We have demonstrated that the prevalence of HPT was related to gender, age, BMI category, and screen time, and the strongest determinant was presence of obesity.

These findings suggest that proactive interventions should be introduced to reduce the prevalence of HPT in this population. Individuals with ID should be included in activities to increase physical activity and reduce weight. These activities should also be addressed to parents or carers of children to raise their awareness about the needs and benefits of lifestyle change in this population with special needs.

The present study indicates significantly lower levels of physical activity among students with ID compared to their peers without ID (1.99 versus 3.02 PAQ score, resp.). Slightly more than 70% of children and adolescents with ID had low physical activity levels, moderate, approx. 30%, and no one had a high level of physical activity. Students without ID were much more active. Nearly 80% of this group had moderate or high levels of physical activity. The results of this study support previous findings. Lin et al. reported that only 8% of adolescents with ID met the national physical activity recommendation in Taiwan which suggests 30 minutes of physical activity at least 3 times a week. They also observed that the increase in the level of ID was accompanied with the significant reduction in the level of physical activity. Physical activity of young people varied depending on the level of parental education and material status. The higher the education and monthly income declared by the parents is, the more often the youth undertook regular physical activity [11]. Emerson's study showed that even fewer (4%) individuals with ID aged 16–24 years in Northern England met criteria for being “physically active” [37]. Mikulovic et al. showed that students with ID spent on average 4.5 hours per week on motor activity and in their peers without ID it was 7.5 hours per week [38]. Other researchers also reported that very few or no children with ID reached recommended levels of physical activity estimated via self-reported questionnaires [39] or accelerometers [40]. Frey et al. used accelerometers to assess the level of physical activity in four different situations: during physical education class, breaks, after school, and weekends. The level of physical activity in the children with ID was significantly lower in every analyzed case compared to the students without ID [41]. In turn, Pan et al. showed that the level of physical activity in the adolescents with ID during physical education class was at a comparable level to the physical activity level of their peers without ID. However, during the break, the students with ID were significantly less active than their peers without ID [12]. According to Einarsson et al., the time spent in moderate to vigorous physical activity among children with ID was only 41%, 50%, and 32% of that of children without ID during entire weekday, school hours, and after school hours, respectively [10]. It was also shown that children with ID are less likely to participate in out-of-school sports than their peers without ID [42, 43]. To overcome the most prominent barriers in taking up physical activity in people with ID (such as health problems, lack of motivation) it is extremely important to start to reduce or eliminate environmental barriers. In addition, healthcare professionals should advise their regular patients to become physically active by taking regular physical activity such as walking, jogging, cycling, swimming, and sports and to improve their fitness for the primary prevention of HPT.

Data on sedentary behavior among the students with ID is ambiguous. The results of our study indicate that the average screen time in the children and adolescents with ID was 3.38 hours a day during school days and 3.85 hours a day during weekends. Among students without ID this time was lower and amounted to 2.27 and 3.58 hours, respectively. Similar results were obtained by Einarsson et al. who reported that non-ID children are 44% more active and 24% less sedentary than children with ID during the entire weekday [10]. Prior studies had also shown that individuals with ID were more likely to lead sedentary lifestyles and were less active than their counterparts without ID [44, 45]. On the other hand, Mikulovic et al. indicated that the average screen time among the adolescents with ID was 26.6 hours per week and was close to that of the peers without ID [38]. Also Foley and McCubbin did not observe the difference in time devoted to watching TV or computer games between ID and non-ID individuals. Time spent on watching TV in children with ID was longer, but not significantly different, than time spent on viewing TV in children without ID [46]. The divergence of the above-mentioned results may be due to differences in the level of ID in the studied individuals. To better understand the sedentary behavior of the individuals with ID, it is necessary to examine a large group across all severity levels of ID.

Our findings raise serious concerns and corroborate the high prevalence of both risk factors of HPT: low physical activity and high screen time among children and adolescents with ID. There is still a need to promote physical activity in childhood and adolescence, and the results of our research may help to develop effective interventions for this population, especially as the topic of physical activity in the individuals with ID is significantly underresearched [47]. There is ample evidence that increase of physical activity has a positive effect on BP. Results of randomized controlled trial of exercise intervention including 60 minutes of physical activity 3 times a week for 3 months showed reduction in office and 24 h ambulatory BP in the physical activity group compared with no intervention. After three months, BP in the intervention group was lower by about 6 mm Hg for clinic SBP and DBP, and 10.7 mm Hg for 24-hour ambulatory SBP. A follow-up after 6 months indicated that the intervention group had improvements in carotid intima-media thickness and arterial stiffness [48]. The results of systematic review and meta-analysis have also confirmed that increased physical activity leads to a significant BP reduction in patients with established HPT [49].

Our study indicated the importance of promoting educational programs on healthy lifestyles among young population with ID. Future research should focus on developing effective strategies and physical activity programs tailored for individuals with ID, since physical activity is of great importance, given the established relationship between physical activity and health.

### 5.1. Limitation

This study is not without limitations. The assessment of the level of physical activity using the self-assessment method (PAQ-C, PAQ-A) can be considered as a study limitation. We decided to use this method, instead of accelerometry-based measures (the golden standard for assessing the level of physical activity), due to a large number of the study and control groups. However, the results of the study indicate that the PAQ-C and PAQ-A are reliable and reproducible tools used to assess physical activity in children and adolescents. In addition, we ensured that questionnaires were properly completed, under the supervision of researchers previously trained for their application.

## 6. Conclusions

Low level of physical activity and long screen time were associated with significantly higher HPT risk among the children and adolescents with ID.

## Figures and Tables

**Table 1:** Detailed characteristics of the groups.

Variable	Study group (with ID)	Control group (without ID)	*p*
Age (years)			
7–13^a^	346 (61.0)	346 (61.0)	1.000
14–18^a^	221 (39.0)	221 (39.0)	
Sex^a^			
Girl	283 (49.8)	283 (49.8)	1.000
Boy	285 (50.2)	285 (50.2)	
Body height percentile^b^	33.99 (31,14)	44.77 (28.88)	<0.001^*∗*^
BMI percentile^b^	55.18 (33.79)	48.41 (31.67)	0.001^*∗*^
SBP (mm Hg)^b^	118.51 (11.22)	115.99 (9.11)	0.001^*∗*^
DBP (mm Hg)^b^	68.79 (5.98)	68.62 (4.32)	0.252
SBP percentile^b^	74.81 (23.67)	69.46 (22.66)	<0.001^*∗*^
DBP percentile^b^	74.35 (17.79)	75.03 (15.50)	0.935
Level of ID^a^			
Mild	278 (24.5)	n/a	n/a
Moderate	239 (21.1)		
Severe	50 (4.4)		
PAQ score^b^	1.99 (0.74)	3.02 (0.81)	<0.001^*∗*^
Level of physical activity^a^			
Low	399 (70.4)	124 (21.9)	
Moderate	168 (29.6)	351 (61.9)	<0.001^*∗*^
High	0 (0.0)	92 (16.2)	
Screen time on school days (h/day)^b^	3.38 (1.66)	2.27 (1.19)	<0.001^*∗*^
Screen time on weekends (h/day)^b^	3.85 (1.48)	3.59 (1.62)	0.016

^a^Mean (SD); ^b^*n* (%); SBP: systolic blood pressure; DBP: diastolic blood pressure; ID: intellectual disability; PAQ: Physical Activity Questionnaire. ^*∗*^Statistically significant results.

**Table 2:** Odds ratio for hypertension prevalence depending on physical activity level.

Classification of blood pressure	Level of physical activity								
	High		Moderate			Low			*p*
	*n*	%	*n*	%	OR (95% CI)	*n*	%	OR (95% CI)	
Study group (with ID)									
Normotension	—	—	125	74.4	(REF)	220	55.1	0.42 (0.28–0,63)	<0.001^*∗*^
Prehypertension	—	—	32	19.0	(REF)	85	21.3	1.15 (0.73–1.81)	0.545
Hypertension	—	—	11	6.5	(REF)	94	23.6	4.40 (2.29–8.46)	<0.001^*∗*^

Control group (without ID)									
Normotension	81	88.0	284	80.9	0.58 (0.29–1.14)	82	66.1	0.27 (0.13–0.55)	0.001^*∗*^
Prehypertension	8	8.7	56	16.0	1.99 (0.91–4.35)	24	19.4	2.52 (1.08–5.90)	0.095
Hypertension	3	3.3	11	3.1	0.96 (0.26–3.51)	18	14.5	5.04 (1.44–17.66)	<0.001^*∗*^

REF: reference group. ^*∗*^Statistically significant results. ID: intellectual disability.

**Table 3:** Odds ratio for hypertension prevalence in the study groups depending on screen time.

Classification of blood pressure	Study group (with ID)				Control group (without ID)			
	Screen time							
	<2 h/day	≥2 h/day		*p*	<2 h/day	≥2 h/day		*p*
	*n* (%)	*n* (%)	OR (95% CI)		*n* (%)	*n* (%)	OR (95% CI)	
Screen time on school days								
Normotension	134 (67.0)	211 (57.5)	0.67 (0.46–0.96)	0.027^*∗*^	231 (80.8)	216 (76.9)	0.79 (0.53–1.19)	0.256
Prehypertension	43 (21.5)	74 (20.2)	0.92 (0.60–1.41)	0.707	46 (16.1)	42 (14.9)	0.92 (0.58–1.45)	0.709
Hypertension	23 (11.5)	82 (22.3)	2.21 (1.34–3.65)	0.002^*∗*^	9 (3.1)	23 (8.2)	2.74 (1.25–6.04)	0.009^*∗*^

Screen time on weekends								
Normotension	68 (69.4)	277 (59.1)	0.64 (0.40–1.02)	0.057	104 (81.9)	343 (78.0)	0.78 (0.47–1.30)	0.339
Prehypertension	20 (20.4)	97 (20.7)	1.02 (0,59–1.74)	0.951	18 (14.2)	70 (15.9)	1.15 (0,65–2.01)	0.634
Hypertension	10 (10.2)	95 (20.3)	2.24 (1.12–4.46)	0.020^*∗*^	5 (3.9)	27 (6.1)	1.60 (0.60–4.23)	0.344

ID: intellectual disability. ^*∗*^Statistically significant results.

**Table 4:** Variables influencing prevalence of hypertension in children and adolescents with ID: multinomial logistic regression (progressive step method).

Hypertension (0—no; 1—yes)	OR (95% CI)	*p*
Sex (0, girl)		
Boy	2.24 (1.35–3.72)	0.002^*∗*^
Age (0—7–13 years)		
14–18 years	3.79 (2.30–6.25)	0.000^*∗*^
Body weight classification (0—healthy weight)		
Underweight	0.32 (0.07–1.40)	0.131
Overweight	1.73 (0.88–3.39)	0.111
Obesity	7.88 (4.38–14.19)	0.000^*∗*^
Screen time on school days (0—<2 h/day)		
≥2 h/day	1.83 (1.02–3.29)	0.044^*∗*^
Screen time on weekends (0—<2 h/day)		
≥2 h/day	2.20 (1.00–4.85)	0.051

^*∗*^Reference groups are the groups indicated in parentheses. The model included age, sex, level of disability, BMI categories, physical activity level, and screen time.
